# Supplementary material for: Muscle Enriched Lamin Interacting Protein (Mlip) Binds Chromatin and Is Required for Myoblast Differentiation
Source: Cells. 2021 Mar 10;10(3):615. doi: 10.3390/cells10030615 (PMC7998221; doi:10.3390/cells10030615)
Supplement: Supplementary file 1 [file cells-10-00615-s001.pdf]

Supplementary materials

Table S1. Mlip mediated immunoprecipitated chromatin sequences and proximate genes.

Seq ID	Chromosome	Start	End	Gene Name (Refseq_Id)
1	chr11	100630562	100631801	Ghdc (NM_031871); Stat5b (NM_011489); Hcrt (NM_010410)
2	chr12	87699569	87701282	1700020O03Rik (NM_027405); Esrrb (NM_011934)
3	chr4	94718170	94720094	Jun (NM_010591)
4	chr11	103792159	103792691	Nsf (NM_008740); Arf2 (NM_007477)
5	chr7	19679683	19680704	Six5 (NM_011383); Dmpk (NM_032418); Fbxo46 (NM_175530)
6	chr17	74298854	74299437	Xdh (NM_011723); Srd5a2 (NM_053188)
7	chr1	136186541	136187377	Myog (NM_031189); Tmem183a (NM_001042485); Adora1 (NM_001039510)
8	chr19	5424222	5425145	Drap1 (NM_024176); Sart1 (NM_016882); AI837181 (NM_134149)
9	chr11	98448048	98448687	Ormdl3 (NM_025661); Zpbp2 (NM_199419); Gsdm3 (NM_001007461)
10	chr11	102211161	102212432	Slc4a1 (NM_011403); Ubtf (NM_011551); Rundc3a (NM_016759)
11	chr12	74146686	74147395	Six1 (NM_009189); Six4 (NM_011382)
12	chr11	51911086	51912012	Cdkl3 (NM_153785); Ppp2ca (NM_019411)
13	chr11	97440016	97440554	E130012A19Rik (NM_175332); P140 (NM_018873)
14	chr6	133984873	133986247	Etv6 (NM_007961)
15	chr13	29043739	29044507	Sox4 (NM_009238)
16	chr8	74998169	74999364	Cherp (NM_138585); 1700030K09Rik (NM_028170); Slc35e1 (NM_177766)
17	chr10	77531835	77532953	Icosl (NM_015790); Dnmt3l (NM_019448); D10Jhu81e (NM_138601)
18	chr11	99370875	99371428	Krt39 (NM_213730); Krt23 (NM_033373)
19	chr11	119405479	119406288	Nptx1 (NM_008730); A730011L01Rik (NM_177394); 4932417H02Rik (NM_028898)
20	chr15	77671713	77672494	Myh9 (NM_022410); Txn2 (NM_019913)
21	chr18	64499119	64499702	St8sia3 (NM_009182); Onecut2 (NM_194268)
22	chr11	71843935	71844719	Aipl1 (NM_053245); 6720460F02Rik (NM_144526)
23	chr11	63525177	63525778	---
24	chr13	83641997	83642986	Mef2c (NM_025282)
25	chr18	11998769	11999295	Cables1 (NM_022021); Rbbp8 (NM_001081223)
26	chr11	116155777	116156708	Exoc7 (NM_016857); Galr2 (NM_010254); Foxj1 (NM_008240)
27	chr13	31898394	31900508	Foxc1 (NM_008592); Gmds (NM_146041)
28	chr14	63863727	63864246	Gata4 (NM_008092); Neil2 (NM_201610)
29	chr2	56966823	56969069	Nr4a2 (NM_013613)
30	chr12	57796678	57798624	Pax9 (NM_011041); Slc25a21 (NM_172577); Nkx2-9 (NM_008701)
31	chr11	74361444	74361993	Garnl4 (NM_001015046); E130309D14Rik (NM_001013784)
32	chr11	117127202	117127890	Sept9 (NM_017380)
33	chr4	40800300	40801433	B4galt1 (NM_022305); Smu1 (NM_021535); Spink4 (NM_011463)

34	chrX	83437391	83438048	Nr0b1 (NM_007430); CN716893 (NM_001033492);
35	chrX	49966819	49967783	Gpc3 (NM_016697)
36	chr15	74785771	74786586	Ly6e (NM_008529); Ly6i (NM_020498); 2010109I03Rik (NM_025929)
37	chr8	28338228	28339098	Adrb3 (NM_013462); Got1l1 (NM_029674); Eif4ebp1 (NM_007918)
38	chr14	73724457	73725035	P2ry5 (NM_175116); Itm2b (NM_008410)
39	chr19	5490409	5491450	Cfl1 (NM_007687); B930037P14Rik (NM_001024560); Mus81 (NM_027877)
40	chr10	86955436	86955989	Ascl1 (NM_008553); Pah (NM_008777)
41	chr11	68504683	68506247	Myh10 (NM_175260); Ccdc42 (NM_177779)
42	chr12	28025952	28027255	Sox11 (NM_009234)
43	chr4	99322713	99324046	Foxd3 (NM_010425); Alg6 (NM_001081264)
44	chr7	146766745	146767789	Nkx6-2 (NM_183248); Inpp5a (NM_183144)
45	chr9	31720468	31721092	Barx2 (NM_013800)
46	chr15	101754616	101755305	Krt4 (NM_008475); Krt79 (NM_146063); Krt76 (NM_001033177)
47	chr1	93697504	93699849	Twist2 (NM_007855)
48	chr18	9957761	9958372	Colec12 (NM_130449); Thoc1 (NM_153552)
49	chr6	128347966	128348494	4933413G19Rik (NM_027697); Nrip2 (NM_021717)
50	chr5	124022660	124023186	Vps33a (NM_029929); Clip1 (NM_019765); Diablo (NM_023232)
51	chr5	42155140	42155663	Nkx3-2 (NM_007524); A230052c04Rik (NM_001081422); Rab28 (NM_027295)
52	chr2	104967444	104968001	Wt1 (NM_144783); 0610012H03Rik (NM_028747)
53	chr11	93816692	93817462	Nme1 (NM_008704); Nme2 (NM_001077529)
54	chr11	96896561	96898285	Lrrc46 (NM_027026); Scrn2 (NM_146027); Mrpl10 (NM_026154)
55	chr11	115719698	115720727	Llgl2 (NM_145438); Recql5 (NM_130454)
56	chr11	19727743	19728296	Spred2 (NM_033523)
57	chr13	60278102	60279401	Gas1 (NM_008086)
58	chr11	95479950	95480528	Ngfr (NM_033217); Phb (NM_008831)
59	chr12	8506083	8507206	Rhob (NM_007483); Slc7a15 (NM_177802)
60	chr11	95040709	95041283	Dlx4 (NM_007867); Tac4 (NM_053093)
61	chr11	120675056	120676177	Fasn (NM_007988); Ccdc57 (NM_027745); Dus1l (NM_026824);
62	chr10	87922177	87922771	Sycp3 (NM_011517); Gnptab (NM_001004164); Chpt1 (NM_144807)
63	chr17	74927855	74928595	Birc6 (NM_007566); Yipf4 (NM_026417)
64	chr15	37162017	37162801	Grhl2 (NM_026496)
65	chr11	117643094	117643805	Tmc6 (NM_181321); Tmc8 (NM_181856)
66	chr15	38230634	38232322	Klf10 (NM_013692)
67	chr11	70265548	70266868	Med11 (NM_025397); Arrb2 (NM_145429); Cxcl16 (NM_023158)
68	chr17	86020825	86021335	Six3 (NM_011381); Six2 (NM_011380)
69	chr18	67800598	67801272	Cep76 (NM_001081073); Spire1 (NM_194355); Psmg2 (NM_134138)
70	chr11	87392045	87392751	Tex14 (NM_031386); Sept4 (NM_011129)
71	chr3	81948799	81950480	Gucy1a3 (NM_021896)
72	chr1	120949506	120950599	Gli2 (NM_001081125)
73	chr11	115825936	115826456	Sap30bp (NM_020483); Recql5 (NM_130454); Itgb4 (NM_001005608)
74	chr12	86809880	86810448	Tmed10 (NM_026775); Fos (NM_010234)
75	chr2	179959087	179960141	Lama5 (NM_001081171); Adrm1 (NM_019822); Rps21 (NM_025587)

76	chr10	76576174	76577066	Col18a1 (NM_009929); Col18a1 (NM_001109991)
77	chr6	52208927	52210108	Hoxa13 (NM_008264); Hoxa11 (NM_010450); Evx1 (NM_007966)
78	chr9	62993518	62994021	Lbxcor1 (NM_172446); Map2k5 (NM_011840)
79	chr2	115889119	115889844	Meis2 (NM_010825)
80	chr5	34529892	34530646	Mxd4 (NM_010753); Zfyve28 (NM_001015039); BC023882 (NM_146159)
81	chr7	151767875	151768526	Fadd (NM_010175); Tmem16a (NM_178642)
82	chr1	59819791	59820862	Nol5 (NM_018868); Bmpr2 (NM_007561)
83	chr17	25707253	25708776	Sox8 (NM_011447); Tmem112 (NM_029624)
84	chr11	4940358	4942017	Ap1b1 (NM_007454); Nefh (NM_010904); Rasl10a (NM_145216)
85	chr11	115674223	115675252	Caskin2 (NM_080643); 2310067B10Rik (NM_028014); Tsen54 (NM_029557)
86	chr7	144510580	144511119	Ebf3 (NM_010096)
87	chr19	7016327	7016862	Bad (NM_007522); Plcb3 (NM_008874); Gpr137 (NM_207220)
88	chr4	120339211	120340424	Cited4 (NM_019563); Kcnq4 (NM_001081142); Ctps (NM_016748)
89	chr11	98608474	98609336	Thra (NM_178060); Nr1d1 (NM_145434); Med24 (NM_011869)
90	chr4	126412930	126413573	Tcfap2e (NM_198960); Psmb2 (NM_011970); Ncdn (NM_011986)
91	chr7	150644921	150647194	Cdkn1c (NM_009876); Kcnq1 (NM_008434); Slc22a18 (NM_008767)
92	chr5	123093716	123094444	P2rx7 (NM_001038887); P2rx7 (NM_011027); P2rx7(NM_001038839): P2rx7 (NM_001038845); Ift81 (NM_009879); P2rx4 (NM_011026)
93	chr11	117151838	117152346	Sept9 (NM_017380)
94	chr6	54922105	54922934	A030007L17Rik (NM_026637); Nod1 (NM_172729)
95	chr11	106916663	106917182	Bptf (NM_176850); Kpna2 (NM_010655)
96	chr3	108217649	108218310	Celsr2 (NM_001004177); Celsr2 (NM_017392); Psrc1 (NM_019976); Sars (NM_011319)
97	chr10	126723023	126723537	Mbd6 (NM_033072); Dctn2 (NM_027151); Ddit3 (NM_007837)
98	chr11	58773501	58775801	Hist3h2a (NM_178218); Trim17 (NM_031172)
99	chr11	100831429	100831962	Ptrf (NM_008986); Stat3 (NM_011486); Atp6v0a1 (NM_016920)
100	chr11	68605382	68606822	Myh10 (NM_175260); Ndel1 (NM_023668)
101	chr7	147129648	147130200	Utf1 (NM_009482); Kndc1 (NM_177261); 6430531B16Rik (NM_001033465)
102	chr15	102552835	102553425	Calcoco1 (NM_026192)
103	chr9	58883036	58883646	Neo1 (NM_001042752); Neo1 (NM_008684)
104	chr11	118268930	118269765	Cant1 (NM_001025617); Cant1 (NM_001025618); Cant1 (NM_029502); Lgals3bp (NM_011150); C1qtnf1 (NM_019959)
105	chr2	25402131	25402797	Traf2 (NM_009422); Edf1 (NM_021519)
106	chr6	66986606	66987224	Gadd45a (NM_007836); Gng12 (NM_025278)
107	chr11	60521418	60523920	Llgl1 (NM_008502); Flii (NM_022009)
108	chr3	109143159	109143979	Vav3 (NM_020505) Sm Mus, Brain
109	chr13	100669858	100670438	Cartpt (NM_001081493) ; Cartpt (NM_013732); Mccc2 (NM_030026)
110	chr4	124330281	124330780	Pou3f1 (NM_011141)

111	chr11	100940818	100941389	Hsd17b1 (NM_010475); Naglu (NM_013792); Coasy (NM_027896)
112	chr16	91225808	91226939	Olig2 (NM_016967); Olig1 (NM_016968)
113	chr11	120672947	120673639	Fasn (NM_007988); Ccdc57 (NM_027745); Dus1l (NM_026824)
114	chr11	115179551	115180565	Otop2 (NM_172801); Ush1g (NM_176847); Fads6 (NM_178035); Otop3 (NM_027132)
115	chr11	96133010	96133623	Hoxb9 (NM_008270); Hoxb13 (NM_008267); Hoxb8 (NM_010461)
116	chr14	56443355	56445575	Nfatc4 (NM_023699); Cbln3 (NM_019820); Ripk3 (NM_019955)
117	chr11	95047034	95047892	Dlx4 (NM_007867); Tac4 (NM_053093)
118	chr11	119099564	119100144	Ccdc40 (NM_175430); Tbc1d16 (NM_172443); Gaa (NM_008064)
119	chr8	109127090	109128309	Cdh1 (NM_009864); Cdh3 (NM_001037809); Tmco7 (NM_173037)
120	chr11	120145451	120146020	Bahcc1 (NM_198423); Actg1 (NM_009609)
121	chr3	52071980	52072824	Foxo1 (NM_019739)
122	chr10	80894130	80894639	Ncln (NM_134009); Nfic (NM_008688)
123	chr12	105711659	105712812	Gsc (NM_010351)
124	chr11	102102458	102103002	Hdac5 (NM_010412); BC030867 (NM_153544)
125	chr9	56920383	56921470	Sin3a (NM_001110351); Ptpn9 (NM_019651); Sin3a (NM_001110350)
126	chr8	94880016	94881431	Irx5 (NM_018826)
127	chr15	76303887	76305767	Scx (NM_198885); Hsf1 (NM_008296)
128	chr3	57378168	57379615	Wwtr1 (NM_133784); Commd2 (NM_175095)
129	chrX	103222340	103222844	Cox7b (NM_025379); Atp7a (NM_001109757)
130	chr14	118634852	118636066	Sox21 (NM_145464)
131	chr13	94269153	94270030	Jmy (NM_021310); Homer1 (NM_152134)
132	chr11	75027252	75027826	Rtn4rl1 (NM_177708); Rpa1 (NM_026653); Dph1 (NM_144491)
133	chr11	118934267	118934799	Cbx4 (NM_007625); Cbx8 (NM_013926)

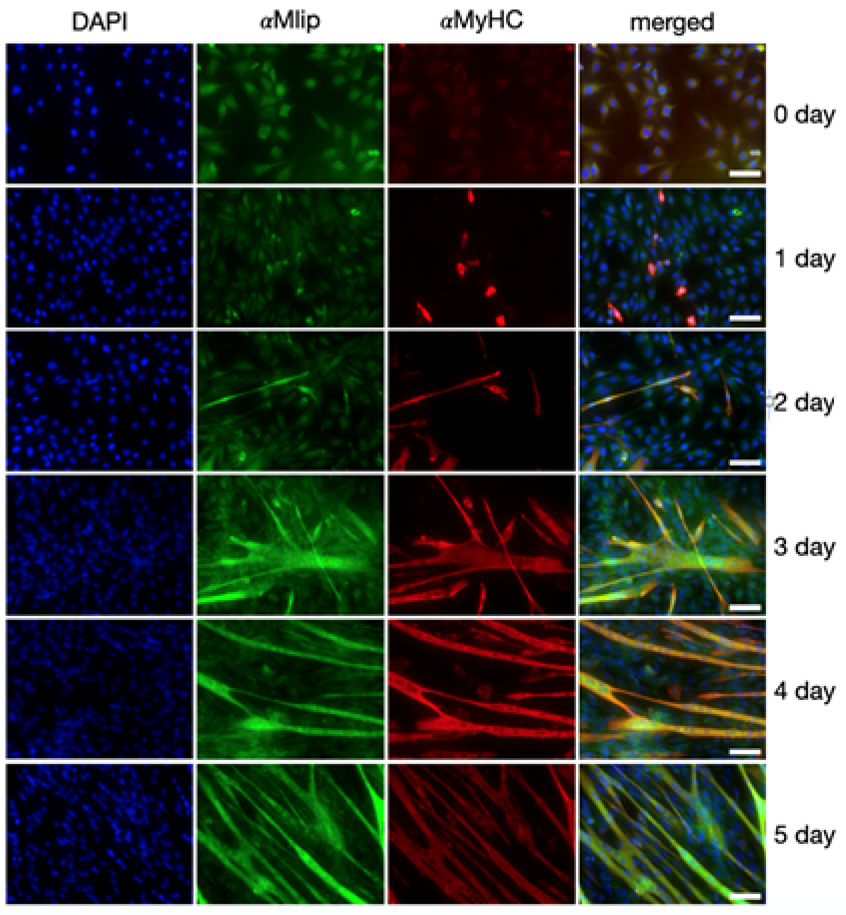

Figure S1. C2C12 differentiation. Indirect immunofluorescence of Mlip (green) and myosin heavy chain (MyHC, red) expression during C2C12 differentiation in the normal or in MLIP-depleted C2C12 myoblasts. Nuclei were stained with DAPI. 20 × magnification; Scale bar = 25 uM.
